# Cultural Adaptations to the Assessment and Treatment of Trauma Experiences Among Racial and Ethnic Minority Groups: A Mixed-Methods Systematic Review and Meta-Analysis

**DOI:** 10.1177/15248380251320982

**Published:** 2025-02-27

**Authors:** Lewis Benjamin, Steve Gillard, Jessica Jones Nielsen, Mariana Costa E. Silva, Jacqueline Sin

**Affiliations:** 1 City St George’s, University of London, UK

**Keywords:** cultural contexts, mental health effects, PTSD, race/ethnicity, trauma

## Abstract

A higher prevalence of post-traumatic stress disorder (PTSD) exists among racial and ethnic minority groups who experience trauma; however, little is known about cultural adaptations of trauma assessments and interventions, or whether those adaptations meet cultural needs. This systematic review examined the effectiveness and experiences of culturally adapted trauma assessments and interventions for adults from racial and ethnic minority groups. Empirical studies investigating culturally adapted trauma assessment and/or interventions targeting adults from racial and ethnic minority groups were searched for in MEDLINE, PsycINFO, Embase, Scopus, CINAHL, Cochrane Central Register of Controlled Trials, Web of Science, from inception to May 2022. A total of 21 articles were included, and 8 common themes of adaptations were identified: socio-cultural integrations, collaboration, psychoeducation, language, cultural matching, addressing stigma, training for providers, and practical considerations. Random effects meta-analyses on intervention effects showed that culturally adapted interventions were more effective in reducing PTSD symptoms (7 randomized controlled trials [RCTs], *n* = 213, Standardized Mean Difference −0.67, 95% CI [−1.06, −0.25], *I*^2^ = 39%) and in ameliorating anxiety symptoms (5 RCTs, *n* = 168, SMD −1.92, 95% CI [−3.18, −0.67], *I*^2^ = 89%) when compared with non-adapted interventions at immediate post-intervention. No statistical difference in effects was found on depression, nor on PTSD or anxiety sustained beyond the post-intervention time-point. Thematic synthesis on participants’ experiences showed that adapted interventions had positive influences on attitudes toward mental health and engagement with services. Future research should employ large-scale trial methods to test adapted trauma interventions over longer follow-up periods as well as to explore the subjective experiences of users of adapted interventions.

## Introduction

Global research has shown that the prevalence of post-traumatic stress disorder (PTSD) in adults ranges from approximately 1% to 12% (NICE, 2018). PTSD is a psychiatric condition that develops following exposure to a traumatic event, with signature symptoms of PTSD including re-experiencing the traumatic event, cognitive disturbance, hyperarousal, and avoidance (American Psychiatric Association, 2013). The Adult Psychiatric Morbidity Survey in the United Kingdom reported higher rates of PTSD among Black British groups compared to White British adults (McManus et al., 2016). Similar findings have been reported in the literature in the United States, suggesting higher rates of PTSD in African American and Hispanic adult populations compared to White groups (Alegría et al., 2013; Asnaani et al., 2010; Roberts et al., 2011; Sibrava et al., 2019).

Complex PTSD (CPTSD) was included as a diagnosis in the 11th revision of the International Classification of Diseases (World Health Organization, 2018). CPTSD encompasses the core features of PTSD as well as further disturbances in emotional regulation, self-concept, and interpersonal relationships. The introduction of CPTSD as a separate diagnostic classification to PTSD was built on extensive clinical observations that showed that those exposed to repetitive or prolonged traumas, such as childhood sexual abuse, often led to complex reactions that extended beyond reactions found in those who experienced trauma following a single event, such as a motor accident (Maercker et al., 2013). Experiences of adverse events such as racism, discrimination, and events related to fleeing persecution or war for refugee and asylum-seeking groups may render vulnerability to ongoing traumatic stressors and greater exposures to trauma (e.g., torture, imprisonment). While this prompts valid inquiry into the prevalence and experiences of CPTSD among racial and ethnic minority groups, studies exploring cultural variations among those with CPTSD remain limited (Heim et al., 2022).

And so, critically understanding the various cross-cultural factors that may shape or influence trauma experiences among racial and ethnic minority groups becomes important. This may include examining sociocultural explanatory models of illness and coping that may influence the interpretation of trauma (Terheggen et al., 2001), as well as trauma disclosure (Long et al., 2007). For example, while those who are exposed to interpersonal abuse may report a heightened sense of anger as a result of injustice following trauma (Erzar et al., 2019; Goldner et al., 2019), religious or spiritual models around forgiveness or revenge may impact this degree of anger, or how it is expressed through cultural norms (Hinton et al., 2003). Similarly, higher rates of stigmatization of mental illness among Black and Asian groups (Alvidrez et al., 2008; Yang, 2007) may prevent help-seeking behavior or disclosing trauma, through fear of backlash from local community members (Laban et al., 2008). In addition, cultural variations in cognitive appraisals and self-concept may have influences on the development and management of trauma (Bernardi et al., 2019).

It is important to acknowledge the role of health care delivery and systems of medical care that currently operate within modern society globally too. To a greater or lesser extent, while most societies involve some form of pluralistic medical system (Johnson et al., 2017), access to, and engagement with healthcare systems vary across diverse cultural and social contexts. This, coupled with an individual’s perception and beliefs (e.g., the anticipation of being misunderstood by statutory health services) will inform how cultural groups engage with services and embark on accessing the right support through these services (Scheppers et al., 2006). Services then failing to acknowledge and meet the cultural needs of service users risk worsening symptoms and outcomes.

Currently, the National Institute for Health and Care Excellence (NICE) guidance for treating PTSD in the United Kingdom includes trauma-focused cognitive behavioral therapy (CBT), prolonged exposure therapy, eye movement desensitization and reprocessing therapy (EMDR), and narrative exposure therapy (NICE, 2018), and several published meta-analyses show positive outcomes of effectiveness for these interventions (Cusack et al., 2016; Davidson & Parker, 2001; Lely et al., 2019; Powers et al., 2010; Seidler & Wagner, 2006). However, it is unclear if the effectiveness of such interventions can be fully generalized across racialized minority groups due to low uptake participating in clinical trials and research, remaining underrepresented at a global level (Brown et al., 2014). Further, clinical guidance on the management of CPTSD and associated difficulties remains outstanding, making it a challenge for clinicians working with individuals presenting with CPTSD or complex trauma presentations. Critics have also cited that evidence-based interventions have mostly been developed in Western contexts (Bernal et al., 2009), arguing that proposed interventions may not be as acceptable to non-Western groups and societies that uphold diverse cultural practices and beliefs around health and illness. Worse, consistent stark racial disparities in outcomes and experiences exist for racial and ethnic minority groups engaging with current healthcare services (Das-Munshi et al., 2018; Hussain et al., 2022). There is therefore a pressing need for global research and services to ensure interventions designed to assess and treat PTSD/CPTSD or trauma experiences are efficacious among diverse service user populations.

For racial and ethnic minority groups, there is some evidence to suggest that culturally adapted interventions can increase intervention effectiveness in public health services (Kalibatseva & Leong, 2014; Kohn et al., 2002) and that cultural groups are more likely to engage with services when they believe their cultural identities and beliefs are accurately reflected in the delivery of treatment (Bhui & Bhugra, 2004). Cultural adaptation refers to “the modification of an intervention protocol to acknowledge language, culture, and context in such a way that it is compatible with the client’s cultural patterns, meanings, and values” (Bernal et al., 2009). Results from several meta-analyses on the effectiveness of culturally adapted interventions in healthcare vary considerably (Anik et al., 2021; Hall et al., 2016; Rathod et al., 2018). Much of the evidence obtained from these reviews evaluated cultural adaptations across interventions designed to address a wide range of psychiatric disorders, notably depressive disorders. Many reviews also fall short of fully describing the nature of the adaptation process, making it hard to infer which components of the intervention approach were efficacious and the relative individual experiences of the adaptations. While research exploring cultural adaptations to trauma treatments among racial minority groups is growing (Ennis et al., 2020; Menon et al., 2023), the scope of reviews has often been limited to exploring adaptations to CBT. Cross-cultural research has questioned the application of CBT when working with diverse groups, citing CBT practices as individualistic and ignoring the wider social and cultural contexts that may influence distress (Hall et al., 2016). While this may justify the need to explore adaptations to CBT when working with varied groups, a more expansive review of adaptations across several treatment modalities that extend beyond CBT is warranted. This strengthens the literature by exploring the effectiveness and appropriateness of the varied treatment modalities, making more informed recommendations for future practice and policy.

Limited research has therefore been conducted on reviewing cultural adaptations to assessments *and* interventions for trauma experiences for racial and ethnic minority groups. Given the higher prevalence of trauma experiences among these groups, in addition to reported poor outcomes in accessing care, systematic exploration of the effectiveness and experiences of cultural adaptations to varied treatment interventions and assessments of trauma for these groups is warranted.

## Aim

To address this gap, we conducted a systematic review of outcomes and experiences of culturally adapted assessments and interventions for trauma experiences among racial and ethnic minority groups. The review aimed to (a) scope the adaptations and/or design features of assessment and interventions for trauma among racial and ethnic minority groups and (b) determine the experiences and effectiveness of adapted or specifically designed interventions for racial and ethnic minority groups with trauma.

## Methods

### Search Strategy

The review protocol was registered prospectively on PROSPERO (Ref.: CRD42022321884). Search terms in this comprehensive review included keywords and identifiers related to our aims, such as “cultural adaptation,” “treatment,” “trauma,” “racial minority,” “CBT,” “EMDR.” A search of empirical studies was performed in seven databases: MEDLINE, PsycINFO, Embase, Scopus, CINAHL, Cochrane Central Register of Controlled Trials, Web of Science, from inception to May 2022.

### Eligibility Criteria

Screening of primary studies was dependent on the following inclusion criteria: empirical studies using quantitative, qualitative, or mixed-method designs, included adults aged 18 years or older from racial and ethnic minority groups, focused on target participants’ experiences and outcomes of culturally-adapted or specifically designed trauma assessments, and/or psychological interventions for trauma experiences (e.g., PTSD, CPTSD, or related symptoms without diagnosis threshold met or specified) and related mental health symptoms (e.g., depression). We excluded articles that did not involve racial or ethnic minority groups, or which did not separate data for racial and ethnic minority participants in their analysis. We excluded studies from the meta-analysis which included existing or overlapping datasets from another study. We also excluded articles that did not include mental health populations or articles that exclusively focused on pharmacological intervention for mental health conditions. Non-pharmacological treatments such as community-based interventions or adapted psychotherapies offer greater insight into the social, cultural, and psychological needs of racialized groups, which is essential to understanding and addressing trauma in these groups.

### Definition of Adaptation

For the purposes of this review, adaptations refer to a direct change, modification, or adjustment to a standardized assessment or intervention to improve its acceptability for a targeted population (Bernal et al., 2009). Additionally, we were also interested in any assessment or intervention approaches that were specifically designed for a targeted population (e.g., an approach that has been developed at the outset to capture the needs of the target audience).

### Screening and Study Selection

All titles and abstracts of articles identified from the searches, and full-text articles of those that met eligibility criteria were independently screened by two reviewers (L.B. and M.C.E.S.). Where discrepancies occurred, these were resolved through wider research team discussion.

### Quality Assessment (Risk of Bias)

Quality assessment of included articles was independently rated by two reviewers (L.B. and M.C.E.S.) using The Mixed Methods Appraisal Tool (MMAT) v2018 (Hong et al., 2018). The tool measures the quality of studies against independent sets of criteria and measures bias across randomized controlled trials (RCTs), non-randomized studies, descriptive studies, qualitative studies, and mixed methods studies. We classified the overall strength of quality for each article as follows: low quality (<40%); medium quality (≥40% and <80%); and high quality (≥80%). We included a descriptive critique of the factors (e.g., clear research question, rationale for methodology, analysis, etc.) that contributed to high, medium, or low-quality studies alongside their percentage scores and overall strength as per guidance (MMAT, Hong et al., 2018) to help infer a wider, comprehensive picture of study quality.

### Data Extraction

Descriptive characteristics of eligible studies were recorded in a data extraction Excel spreadsheet, including authors, year of publication, country, study design, target populations, trauma type, assessment/intervention and adaptation details, sample size, comparator (if applicable), results (including qualitative data from participants, means and standard deviations for symptom measure scores at several times points if applicable), and conclusions.

### Data Analysis and Synthesis

The synthesis of data comprised three stages. The first stage involved a narrative synthesis of data pertaining to study designs and approaches used in the assessment and/or intervention for trauma.

The second stage of synthesis involved describing cultural adaptations used across intervention and assessment studies, using a narrative approach described by Popay et al. (2006). Identified mechanisms of action were compared between these studies to find the commonalities and differences of certain adaptations and themes that emerged from the data. Any data that accounted for experiences of interventions or assessments was also explored.

Third, meta-analyses were conducted to examine the intervention effects of culturally adapted interventions on PTSD symptoms and common co-morbid difficulties (i.e., depression and anxiety symptoms). Only RCTs that reported symptom outcomes using validated measures were included in the meta-analyses (Concato et al., 2000). We were also interested in outcome measures that may capture complex trauma symptoms (e.g., negative self-concept), which may or may not include measurements of CPTSD. We conducted meta-analyses using the random effects model whenever there were four or more studies reporting usable data to capture any uncertainty resulting from heterogeneity among studies. When data were available from less than four studies, the fixed-effect model was used instead (Borenstein et al., 2010). No subgroup analyses to examine the extent of assessment and intervention effects across certain cultural groups were pre-specified considering the limited volume of data to date. Review Manager software was used to conduct the meta-analyses (RevMan 5.4, provided by Cochrane Collaboration). Standardized mean differences with 95% confidence intervals were calculated for all comparisons with continuous measures. *I*^2^ statistic depicted the heterogeneity (Borenstein et al., 2009): high heterogeneity was assumed if the *I*^2^ value was >75%; moderate with *I*^2^ between 25% and 75%; and low if <25% (Higgins et al., 2003). Chi-square (χ^2^) values were calculated to depict differences between observed and expected frequencies of outcomes and Tau-squared (τ^2^) values were used to indicate estimates of between-study variance. Results of the remaining intervention studies which were not suitable for meta-analyses were narratively described.

## Results

**Figure 1.**
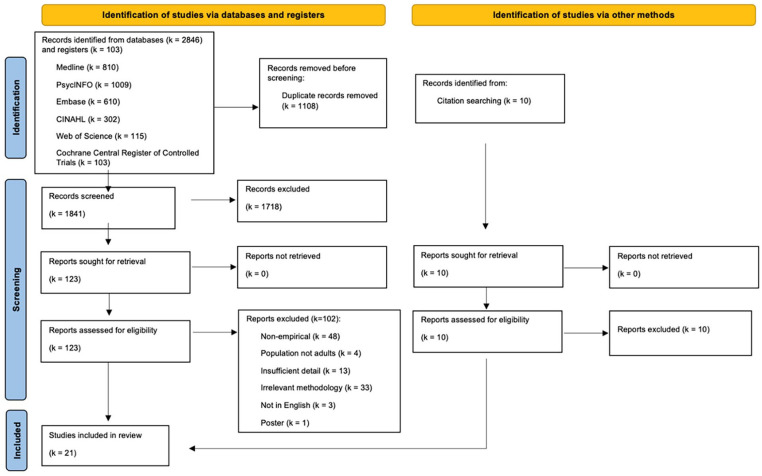
PRISMA flowchart. Overview of database searches and included studies.

A total of 2,949 records were found following the search across databases and following removal of duplicate articles, a total of 1,841 articles remained for title and abstract screening. After screening title and abstracts, we read 123 full-text articles. Full-text screening allowed for a total of 21 eligible articles included in the current review. Figure 1 describes a PRISMA flowchart (Page et al., 2021) and overview of the screening process.

### Overview of Studies

A total of 21 studies with 3,341 adult participants were included in this review. Sample sizes ranged from 7 to 1,358. A detailed description of study characteristics is included in Table 1. In reference to the location of studies, the majority were conducted in the United States (Galano et al., 2017;Hahm et al., 2019; Hinton et al., 2004, 2005, 2011, 2013; James & Noel, 2013; Pearson et al., 2019). Two studies were conducted in Australia (Fernandes & Aiello, 2018; Gibson et al., 2021), one in the Caribbean (Vera et al., 2022), one in Germany (Kananian et al., 2017), one in Pakistan (Latif et al., 2021), two in South Africa (Jalal et al., 2020; Madigoe et al., 2017), two in countries across Southeast Asia (Chhim, 2012; Eichfeld et al., 2019), one in Turkey (Eskici et al., 2021), one in Uganda (Goninon et al., 2021), and two in the United Kingdom (Bahu, 2019; Hammad et al., 2020).

**Table 1.** Overview and Quality Assessment of Included Studies.

Assessment/Intervention Approach	Study Author(s), Year, Country	Study Design	Comparator	Population	Trauma Type	Sample Size, Gender (% of Sample)	Intervention Details (Modality, Duration, Delivery)	Mean Age of Sample (SD)	MMAT Study Quality
Intervention	Bahu (2019), United Kingdom	Mixed methods	None	Tamil refugees	Family or individual experience of imprisonment, torture, arrest, or death	16 Men: 10 (60%) Women: 6 (40%)	CA-CBT, 12 group sessions	32 (NR) Range: 23–55	High
Assessment	Chhim (2012), Cambodia	Mixed methods	N/A	Cambodians	Development group: victims of torture, healthcare, and academic professionals, community leaders Validation group: Stampede victims	Development sample: 53 Men: 34 (64.2%) Women: 19 (35.8%) Validation sample: 390 Men: 122 (31.2%) Women: 268 (68.8%)	N/A	Development sample: 56 (12.87) Validation sample: 53.40 (12.96)	Medium
Intervention	Eichfeld et al. (2019), Indonesia, Cambodia, and Thailand	Pre-post	Control group from another study	Southeast Asians	Natural disaster victims (Indian Ocean Tsunami), interpersonal violence victims, Khmer Rouge regime victims	1,358 Men: 484 (34.5%) Women: 885 (65.2%) Transgender: 5 (0.4%)	Individual and group focused trauma-focused therapy	31.69 (12.02)	High
Intervention	Eskici et al. (2021), Turkey	RCT	TAU	Women Syrian Refugees	NR	23 Women: 23 (100%)	CA-CBT 7 Weekly group sessions, 1.5–2 hr	35.1 (8.3)	High
Intervention	Fernandes and Aiello (2018), Australia	Qualitative	None	Tamil refugees	Male survivors of torture and rape	42 Men: 42 (100%)	Narrative Exposure Therapy and Psychoeducation Group sessions: 10 weekly groups, 2.5 hr Individual sessions: 1–6	NR (NR) Range: 24–49	Low
Intervention	Galano et al. (2017), United States	Controlled study	Waitlist control	Latina immigrant women	Interpersonal violent victims	93 Women: 93 (100%)	Mixed modalities, 10-week group	35.66 (7.2)	High
Intervention	Gibson et al. (2021), Australia	Quasi-experimental controlled	UC/TAU	Tuvaluans	Natural disaster	99 Funafutti sample: Men: 12 (24%) Women: 38 (76%) Nui sample: Men: 21 (43%) Women: 28 (57%)	Psychoeducation: 6 group sessions, across 5 days	Funafutti location: 34.12 (range 18–71) Nui location: 50.02 (range 20–74)	High
Intervention	Goninon et al. (2021), Uganda	Quasi-exp	Immediate, delayed treatment	Congolese refugees	Refugees and victims of war	98 Men: 85 (49%) Women: 89 (51%)	Culturally-adapted trauma focused CBT, 9 group sessions, 1.5 hr	33.4 (11.7)	Medium
Intervention	Hahm et al. (2019), United States	RCT	Waitlist control	Asian American women	Interpersonal trauma	63 Women: 63 (100%)	Mixed modalities 8-Week group and SMS messages	23.63 (NR)	High
Intervention	Hammad et al. (2020), United Kingdom	Mixed methods	None	British MENA-origin Muslim women	Victims of Grenfell fire tragedy	16 Women: 16 (100%)	Mixed modalities First group: 8 weekly 2 hr sessions Second group: 9 weekly 1.5 hr sessions	First group range: 36–83 (NR) Second group range: 41–84 (NR)	High
Intervention	Hinton et al. (2004), United States	Pilot RCT	Immediate treatment, delayed treatment	Vietnamese refugees	Ex-political and non-ex-political detainees	12 Men: 6 (50%) Women: 6 (50%)	CBT, 11 weekly individual sessions	NR (NR)	Medium
Intervention	Hinton et al. (2005), United States	RCT	Initial, delayed	Cambodian refugees	Victims who “passed through Cambodian genocide (1995–1979)”	40 Men: 16 (40%) Women: 24 (60%)	CBT, 12 weekly individual sessions	Initial: 50.90 (6.11) Delayed: 52.70 (7.43)	High
Intervention	Hinton et al. (2011), United States	Pilot RCT	Active treatment control (applied muscle relaxation)	Latina women	NR	24 Women: 24 (100%)	CA-CBT 14 Weekly group, 1 hr	Intervention: 47.6 (8.2) Control: 51.4 (5.9)	High
Assessment	Hinton et al. (2013), United States	Cross-sectional	N/A	Cambodian refugees	All patients “lived through Pol Pot genocide”	226 Men: NR Women: 149 (66%)	N/A	52.6 (9.5)	Medium
Intervention	Jalal et al. (2020), South Africa	Pilot RCT	Active treatment (applied muscle relaxation)	Indigenous South Africans	NR	20 Men: 5 (25%) Women: 15 (75%)	CA-CBT, 14 individual sessions, 7 weeks (2 sessions per week), 1 hr	28.2 (NR)	High
Intervention	James and Noel (2013), United States	Pre-post	None	Haitians	Haiti earthquake survivors	113, NR	Psychoeducation; Lay worker group intervention: 1.5 hr, 3× week for 2 months	Study 1: 35.44 (10.38) Study 2: 39.92 (0.48) Study 3: NR (NR)	Low
Intervention	Kananian et al. (2017), Germany	Pre-post	N/A	Afghan and Iranian refugees	Imprisonment, torture	7 Men: 9 (100%)	CA-CBT, 12 group sessions, once a week, 1.5 hr	25.6 (9.0)	Medium
Intervention	Latif et al. (2021), Pakistan	Feasibility RCT	Waitlist control	Pakistani women	Interpersonal trauma; domestic violence victims	50 Women: 50 (100%)	CA-CBT and GSH, 9 sessions over 12 weeks, 15–20 min	Intervention 27.4 (4.6) Control 26.3 (3.7)	High
Assessment	Madigoe et al. (2017), South Africa	Cross-sectional	N/A	South African Zulu	NR	100 Men: 50 (50%) Women: 50 (50%)	N/A	NR (NR) Range: 18–61	Medium
Intervention	Pearson et al. (2019), United States	RCT	Waitlist control	Native American women	Interpersonal trauma	73 Women: 73 (100%)	CA-CPT, 13 sessions	18–29: (*n* = 16, 21.9%) 30–39: (*n* = 21, 28.8%) 40–49: (*n* = 23, 31.5%) 50–60: (*n* = 13, 17.8%)	High
Intervention	Vera et al. (2022), Puerto Rico	RCT	Active treatment control (applied muscle relaxation)	Latino	Interpersonal violence, unexpected death, transportation accidents, war, and community violence	98 Total sample: Men: 18 (18.4) Women: 80 (81.6)	CA^-^Prolonged exposure, 12–15 individual weekly sessions, 1.5 hr	43.6 (NR)	High

*Note*. CA = culturally adapted; CBT = cognitive behavioral therapy; CPT = cognitive processing therapy; GSH = guided self-help; MENA = Middle East and Northern Africa; MMAT = Mixed Methods Appraisal Tool; N/A = not applicable; NR = not reported; RCT = randomized controlled trial; SMS = short message service; TAU = treatment as usual; UC = uncontrolled.

Study populations were mixed; however, a notable number of studies focused on refugee or asylum-seeking populations from racial and ethnic minority backgrounds (Bahu, 2019; Eskici et al., 2021; Fernandes & Aiello, 2018; Goninon et al., 2021; Hinton et al., 2004, 2005, 2013; Kananian et al., 2017). In terms of study designs, nine studies used randomized controlled study designs (Eskici et al., 2021; Hahm et al., 2019; Hinton et al., 2004, 2005, 2011; Jalal et al., 2020; Latif et al., 2021; Pearson et al., 2019; Vera et al., 2022). One study used a qualitative design (Fernandes & Aiello, 2018), three were mixed methods studies (Bahu, 2019; Chhim, 2012; Hammad et al., 2020), three studies used pre-post evaluation designs (Eichfeld et al., 2019; James & Noel, 2013; Kananian et al., 2017), two studies used quasi-experimental designs (Gibson et al., 2021; Goninon et al., 2021), two studies were cross-sectional studies (Hinton et al., 2013; Madigoe et al., 2017), and one study used a controlled study design (Galano et al., 2017).

### Quality Assessment of Studies

Most articles in the review (Bahu, 2019; Chhim, 2012; Eichfeld et al., 2019; Eskici et al., 2021; Galano et al., 2017; Gibson et al., 2021; Goninon et al., 2021; Hahm et al., 2019; Hammad et al., 2020; Hinton et al., 2004, 2005, 2011, 2013; Jalal et al., 2020; Kananian et al., 2017; Latif et al., 2021; Madigoe et al., 2017; Pearson et al., 2019; Vera et al., 2022) were assessed to have high or medium-high quality (≥40%). Justifications for high-quality rating included having complete outcome data, using appropriate sampling methods to address the research question(s), evaluating response rates, and blinding of outcome assessors (and several more criteria set out in the MMAT). However, a small number of articles (Fernandes & Aiello, 2018; James & Noel, 2013) had low methodological quality (≤40%). Common reasons for this included a lack of complete outcome data, groups not being comparable at baseline, or outcome assessors not being blinded to interventions in RCT studies. See Table 1 for quality ratings of each study.

### Characteristics of Intervention Studies

While studies often included multi-modal modalities in the treatment approach, the most often modality incorporated among most studies was a form of culturally adapted cognitive behavioral approaches (*k* = 15). Two articles (Gibson et al., 2021; James & Noel, 2013) solely focused on psychoeducation. Many studies delivered interventions through group formats (*k* = 12). Duration of the interventions ranged from 7 to 15 weeks with a mean of 11 weeks. A large proportion of intervention studies focused on experiences of refugee and asylum-seeking populations (Bahu, 2019; Eskici et al., 2021; Fernandes & Aiello, 2018; Goninon et al., 2021; Hinton et al., 2004, 2005; Kananian et al., 2017), which included traumas associated with war, forced displacement, imprisonment, torture, and rape.

### Characteristics of Assessment Studies

Three studies focused on evaluating culturally specific assessments for trauma experiences (Chhim, 2012; Hinton et al., 2013; Madigoe et al., 2017). Assessment studies focused on the integration of culturally specific experiences, practices, and knowledge into the development of an assessment tool that assesses and screens for trauma. Two articles (Chhim, 2012; Hinton et al., 2013) focused on assessing trauma experiences among Cambodian populations, and one article (Madigoe et al., 2017) focused on the development of a culturally specific screening tool for trauma among South African Zulu groups. Trauma experiences that were investigated in assessments were therefore locally and culturally determined, which included integration of culturally relevant somatic complaints into the acknowledgment of trauma (Chhim, 2012; Hinton et al., 2013) and an awareness of explanatory models and beliefs around illness (Chhim, 2012; Hinton et al., 2013; Madigoe et al., 2017). Two assessment studies reported the integration of spirituality into the assessment of trauma. Specifically, this included screening for trauma experiences or coping behaviors related to witchcraft or traditional rituals (e.g., interpretation of sleep paralysis and nightmares as spiritual attacks, Hinton et al., 2013). Assessment studies involved comparisons to Western diagnostic screening tools for trauma, with two articles (Chhim, 2012; Hinton et al., 2013) focusing on the PTSD checklist (Civilian Version) and one article (Madigoe et al., 2017) focusing on the PTSD section of the DSM Structured Clinical Interview to inform further validation and comparison of adapted measures.

### Cultural Adaptations

Eight themes emerged on cultural adaptations used targeting racial and ethnic minority groups, and these are depicted below. Further details of these themes are detailed in Table 2 as well as narrative below.

**Table 2.** Summary of Adaptations.

Cultural Adaptation	Examples
Language	Adapting language within assessments or interventions to align with cultural nuances and meanings that are relative to groups. Avoiding Western concepts, terminology, and clinical jargon. Using culturally relevant metaphors, analogies, idioms, and practices to explain trauma. Facilitation of assessments or interventions in the preferred local language.
Sociocultural Considerations	Utilizing methods that harness cultural context and identity and integrate this into practice (e.g., collective cooking/drumming to foster connection and belonging, assessing spirituality to understand meaning-making and coping mechanisms).
Training for Providers	Training for service providers that understands the cultural, social, and historical contexts of the communities they serve to best meet cultural needs.
Psychoeducation	Involves the use of education around trauma experiences in a way that is familiar or relative to the cultural background. Adaptations draw on cultural history and contextual examples to explain trauma experiences and the process of treatment.
Practical considerations	Pragmatic and logistical adaptations to interventions to ensure they are accessible and retain engagement. Offering more trauma sessions devoted to trust building. Offering care where appropriate within existent community networks and settings outside of medical and traditional services (e.g., faith-based places of worship).
Stigma	Reframing interventions to distance from Western concepts, such as “mental health intervention.” Harnessing group discussions that normalize trauma responses, behavior, and provide collective support. An awareness of spirituality and how this may influence trauma responses, such as viewing trauma as spirit possession or punishment.
Collaboration	Utilizing the knowledge and experiences existent within community networks of racial and ethnic minority groups to develop and conduct assessments and interventions, such as input from traditional healers.
Cultural matching	The choice for alignment between therapist’s and individuals’ cultural backgrounds enhances a stronger therapeutic alliance.

#### Language

Language adaptations were reported across intervention and assessment studies. This included the translation of an original intervention or assessment into the local language of participants, through either translated intervention materials (Bahu., 2019; Galano et al., 2017; Goninon et al., 2021; Hammad et al., 2020; Hinton et al., 2004; Madigoe et al., 2017; Pearson et al., 2019) and/or the intervention or assessment in the preferred local language delivered by the facilitator or use of an interpreter (Chhim, 2012; Fernandes & Aiello, 2018; Galano et al., 2017; Hammad et al., 2020; Madigoe et al., 2017). Changing jargon into more culturally specific and understood terminology to improve the acceptability of interventions was also reported across several articles (Hammad et al., 2020; Hinton et al., 2013; Vera et al., 2022).

#### Cultural Matching of the Facilitator

Adaptations to the intervention through cultural matching of the facilitator to participants were present in over a third of all studies (*k* = 8). Cultural matching of the facilitator included matching facilitators and participants for ethnicity as well as other characteristics such as gender or language as a way of improving acceptability of the intervention (Bahu, 2019; Eichfeld et al., 2019; Eskici et al., 2021; Goninon et al., 2021; Hahm et al., 2019; Hammad et al., 2020; James & Noel, 2013; Vera et al., 2022). Studies that included matching cultural backgrounds to participants discussed the benefits of building therapeutic alliance (Hahm et al., 2019; Hammad et al., 2020), as well as rapport and trust (Bahu, 2019; Hammad et al., 2020; James & Noel, 2013). This was particularly notable in studies that included participants with histories of interpersonal traumas whereby participants and facilitators were matched on demographics such as ethnicity and gender. All articles that included cultural matching between facilitators and participants reported training and/or supervision of facilitators to ensure cultural competency is achieved as well as intervention fidelity.

#### Sociocultural Integrations

Most studies included integration of sociocultural considerations into interventions or assessments (*k* = 19). This included ensuring intervention activities were sensitive to participant cultural contexts (Bahu, 2019; Eichfeld et al., 2019; Eskici et al., 2021; Fernandes & Aiello, 2018; Goninon et al., 2021; Hammad et al., 2020; Hinton et al., 2004, 2005, 2011; James & Noel, 2013; Kananian et al., 2017; Pearson et al., 2019; Vera et al., 2022) as well as the integration of cultural values, beliefs, experiences, and practices into assessments or interventions (Bahu, 2019; Chhim, 2012; Eichfeld et al., 2019; Eskici et al., 2021; Fernandes & Aiello, 2018; Goninon et al., 2021; Hahm et al., 2019; Hammad et al., 2020; Hinton et al., 2004, 2005, 2011, 2013; Jalal et al., 2002; James & Noel, 2013; Kananian et al., 2017; Latif et al., 2021; Madigoe et al., 2017; Vera et al., 2022). The integration of faith and spirituality was highlighted in nine articles (Bahu, 2019; Eskici et al., 2021; Hammad et al., 2020; Hinton et al., 2011, 2013; Jalal et al., 2020; James & Noel, 2013; Kananian et al., 2017; Madigoe et al., 2017), with many incorporating prayer and/or religious rituals in interventions, or exploring beliefs held around spirituality in assessment. Culturally specific intervention activities were reported in articles that involved aspects of collective prayer (Fernandes & Aiello, 2018; Hammad et al., 2020), drumming and singing (e.g., darbuka drumming, Nasheed singing, Hammad et al., 2020), and pranayama breathing (Bahu, 2019).

Most articles integrated culturally relevant metaphors, idioms, and concepts into intervention delivery (e.g., Buddha’s arrow and the Chakra system, Bahu, 2019). Normalization of symptoms was also reported using culturally resonant concepts (e.g., parables and vignettes from cultural texts of cultural heroes who experienced trauma, Fernandes & Aiello, 2018). Culturally relevant visualizations aided intervention exercises in many articles too (e.g., breathing and relaxation exercises, Bahu, 2019; Hammad et al., 2020; Hinton et al., 2004, 2005, 2011; Jalal et al., 2020; Kananian et al., 2017).

Several adaptations were made to organize sessions to boost acceptability of cultural groups. Examples included groups set up to resemble social gatherings similar to participants’ native countries and restore culturally familiar ways to participants through cooking traditional group meals with collective storytelling practices (Hammad et al., 2020).

#### Psychoeducation

Almost half of articles (*k* = 8) involved psychoeducation on PTSD and trauma symptoms (Bahu, 2020; Eskici et al., 2021; Hammad et al., 2020; Hinton et al., 2004, 2005, 2011; Kananian et al., 2017; Latif et al., 2021). Unsurprisingly, most of these articles involved adaptations to CBT, where psychoeducation is a key ingredient in CBT treatment. Culturally relevant games were also described in articles to provide psychoeducation around somatic symptoms (e.g., “Kursi Karasi—musical chairs,” Eskici et al., 2021). Traditional folklore or oral storytelling (e.g., “Hikaya,” Hammad et al., 2020; story of Mahatma Gandhi, Bahu, 2019; and Elephant and Six Blind Men to explain exposure, Latif et al., 2021) was integrated into psychoeducation in articles. Culturally appropriate visualizations and imagery such as a lotus bloom to encode key Asian cultural values of flexibility (Hinton et al., 2004, 2005; Kananian et al., 2017) were also utilized to promote discussion and psychoeducation among participant groups.

#### Collaboration

Another theme emerged from the findings was collaboration, where over half (*k* = 11) of articles either involved community stakeholders in the delivery of interventions (Bahu, 2019; Eskici et al., 2021; Galano et al., 2017; Gibson et al., 2021; Hammad et al., 2020; Kananian et al., 2017; Pearson et al., 2019; Vera et al., 2022) and/or involved consultation with participants (Bahu, 2019; Chhim, 2012; Hahm et al., 2019; Hammad et al., 2020; Kananian et al., 2017; Madigoe et al., 2017; Vera et al., 2022). By understanding the social support structures of varied cultural groups, modifications were made to allow support through existing community networks in several articles. Specific examples included liaison with local faith-based community leaders to inform approaches (Bahu, 2019; Chhim, 2012; Hammad et al., 2020), collaborating with community leaders to host interventions outside of mental health settings such as in a community center (Hammad et al., 2020) or local temple (Bahu, 2019), and providing consultation with community stakeholders to develop treatment manuals (Pearson et al., 2019). In one article (Pearson et al., 2019), an additional pre-session dedicated to engagement and rapport building was added to the treatment manual based on community input and consultation between community stakeholders and participants. Similarly, another study (Vera et al., 2022) reported the opportunity for participants to attend a pre-session dedicated to building trust and offered opportunities for participants to bring along a trusted relative to aid support to partake in the intervention.

#### Practical Considerations

A large proportion of intervention articles discussed making practical adaptations (*k* = 12). Some included adapting the time or length of interventions (Eskici et al., 2021; Goninon et al., 2021; Hammad et al., 2020; Kananian et al., 2017; Vera et al., 2022), and/or location of treatment (Hammad et al., 2020; Hinton et al., 2011; Latif et al., 2021).

Modifications to treatment manuals included adapting content to acknowledge the beliefs and experiences of the relevant cultural group. This included content in manuals to refer to Afghan culture with visualization exercises in one study (Kananian et al., 2017) or removing specific jargon and replacing content with relative cultural examples in others (Eskici et al., 2021; Hammad et al., 2020; Hinton et al., 2011). Of those reporting practical considerations, a high proportion of studies referenced an emphasis on encompassing a sensorial or somatic element to interventions (Bahu, 2019; Fernandes & Aiello, 2018; Hammad et al., 2020; Hinton et al., 2011; James & Noel, 2013; Kananian et al., 2017; Pearson et al., 2019). A notable number of these articles included participants from Asian and Hispanic populations as well as from refugee and asylum-seeking populations.

Several articles that focused on trauma experienced by refugee and asylum-seeking groups (Eskici et al., 2021; Goninon et al., 2021; Kananian et al., 2017) referenced high dropout rate of refugees in treatments due to unsettlement in prior literature and adapted sessions accordingly which included fewer sessions with longer time devoted to each session. Other studies (Hammad et al., 2020; Vera et al., 2022) reported longer periods of engagement sessions to address mistrust of services.

#### Training for Providers

A small number of articles (*k* = 6) reported training for providers of interventions (Bahu, 2019; Eichfeld et al., 2019; Eskici et al., 2021; Gibson et al., 2021; Goninon et al., 2021; James & Noel, 2013). Articles discussed how training allowed facilitators to ascertain treatment fidelity and to understand the cultural needs of participants. Of these six articles, four studies (Bahu, 2019; Eichfeld et al., 2019; Eskici et al., 2021; Goninon et al., 2021) involved providers who were mental health professionals, and the remaining two studies involved “non-specialist” peer lay workers (Gibson et al., 2021; James & Noel, 2013). Only two articles reported the time spent devoted to training providers (e.g., 1-week training course, Eskici et al., 2021; 3 months of training, Goninon et al., 2021) with the remaining articles not providing this information. It is also unclear what training included, details of any models or theories underpinning this, as well as who provided the training as only one articles reported this detail (Eskici et al., 2021). This included training on basic helping skills, psychological ﬁrst aid, mitigating challenges in group therapies, and ensuring fidelity to the intervention protocol.

#### Stigma

Three articles explicitly referred to addressing stigma within interventions (Bahu, 2019; Hammad et al., 2020; Jalal et al., 2020). Examples of spirituality were referenced to address cultural stigma in one article (e.g., trauma believed to be a sign of spirit possession, Jalal et al., 2020). Another article details how labeling the intervention as a “non-mental health intervention” was important to tackle the stigma and shame often attached to trauma in Muslim communities (Hammad et al., 2020). Collective discussions between participants around addressing stigma were also utilized (e.g., harnessing the knowledge of the community to overcome stigma, Bahu, 2019).

### Intervention Effects

A total of seven RCTs (Eskici et al., 2021; Hinton et al., 2004, 2005, 2011; Jalal et al., 2020; Pearson et al., 2019; Vera et al., 2022) were included in the meta-analysis to evaluate the effectiveness of culturally adapted trauma interventions on PTSD severity. Five trials looked at PTSD with co-morbid depression outcomes (Hahm et al., 2019; Hinton et al., 2011; Jalal et al., 2020; Latif et al., 2021; Vera et al., 2022). Co-morbid anxiety outcomes were also reported in five trials (Hinton et al., 2004, 2005, 2011; Jalal et al., 2020; Vera et al., 2022).

Of all the RCTs included in the meta-analyses, five RCTs utilized waitlist control comparisons (Hahm et al., 2019; Hinton et al., 2004, 2005; Latif et al., 2021; Pearson et al., 2019), three used active treatment controls (Hinton et al., 2011; Jalal et al., 2020; Vera et al., 2022), and one study (Eskici et al., 2021) used treatment as usual as a comparator. It is worth noting that for the three trials that used active treatment controls all three utilized applied muscle relaxation.

**Figure 2.**
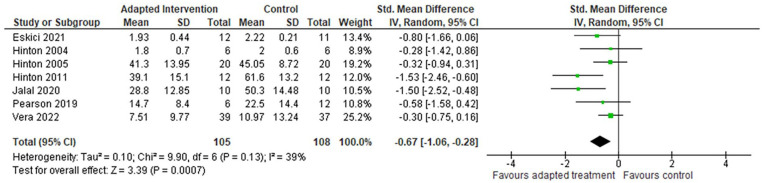
Meta-analysis on participant’s PTSD severity at post-intervention. *Note*. PTSD = post-traumatic stress disorder.

Meta-analyses results comparing culturally adapted intervention groups with all controls for outcomes on PTSD severity showed the former more effective in ameliorating PTSD symptoms at post-intervention (7 RCTs, *n* = 213, SMD −0.67, 95% CI [−1.06, −0.25], *I*^2^ = 39%). Figure 2 presents the forest plot on PTSD severity at post-intervention. Fewer studies reported outcome results at a 3-month follow-up timepoint, and the meta-analysis results found the post-intervention effects on PTSD symptoms no longer sustained (4 RCTs, *n* = 175, SMD −0.38, 95% CI [−0.93, 0.18], *I*^2^ = 67%) (see Supplemental Material).

Findings comparing culturally adapted interventions targeting depression severity outcomes showed no significant effect at post-intervention (5 RCTs, *n* = 206, SMD −1.07, 95% CI [−2.25, 0.11], *I*^2^ = 92%). No follow-up data was available on depression outcomes.

**Figure 3.**
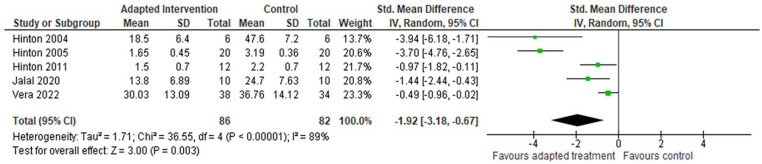
Meta-analysis on participants’ anxiety severity at post-intervention.

Culturally adapted interventions were found to be superior in reducing anxiety symptoms when compared to control groups at post-intervention (5 RCTs, *n* = 168, SMD −1.92, 95% CI [−3.18, −0.67], *I*^2^ = 89%). Figure 3 presents a forest plot on anxiety severity at post-intervention. A fixed effect meta-analysis was run for anxiety severity at 3-month follow up and no significant difference in effects on anxiety severity was found between culturally-adapted interventions and controls (3 RCTs, *n* = 130, SMD −0.26, 95% CI [−0.79, 0.28], *I*^2^ = 53%) (see Supplemental Material).

It is worth noting that high levels of heterogeneity were found in the comparisons of depression and anxiety symptom severity outcomes listed above (*I*^2^ > 75%). High heterogeneity may be accounted for by differences in populations (e.g., some trials targeted same-gender participants), others included varied participants exposed to different trauma experiences. Intervention modalities between trials also differed as detailed in the intervention characteristics above.

#### Effectiveness Results From Non-RCTs

Six quantitative intervention articles and one mixed methods article which measured PTSD severity did not use an RCT design (Eichfeld et al., 2019; Galano et al., 2017; Gibson et al., 2021; Goninon et al., 2021; James & Noel, 2013; Kananian et al., 2017) or included no comparable group (Bahu, 2019).

Consistent with the meta-analysis results, all these intervention articles (*k* = 7) reported a reduction in PTSD symptoms immediately post-intervention for groups receiving culturally adapted interventions. Four of these articles (Bahu, 2019; Gibson et al., 2021; Goninon et al., 2021; James & Noel, 2013) reported a statistically significant reduction in PTSD symptoms at post-intervention (*p* < .05). Little data was reported on co-morbid anxiety and depression severity or follow-up data across non-RCT study designs.

### Participant Experiences

In terms of participant experiences of adapted trauma interventions, two studies explored this using mixed methods (Bahu, 2019; Hammad et al., 2020) and one further study using a qualitative design (Fernandes & Aiello, 2018). The qualitative study involved reflective experiences in a therapy group. No articles explored experiences of adapted trauma assessments.

Two of the intervention articles included target populations from refugee groups; one article (Fernandes & Aiello, 2018) focused on an adapted intervention to support male refugee survivors of rape and torture, and the other article (Bahu, 2019) focusing on trauma related to forced migration among Tamil refugee and asylum-seeking groups. Participants reported positive benefits from attending the interventions, particularly reporting reduced shame related to trauma following attendance, as well as more confidence navigating the refugee and immigration process. Qualitative data highlighted how stigma and shame had previously impacted participants’ help-seeking behaviors as well as knowledge around trauma and mental health, and how adapted interventions had positively changed attitudes toward therapy and accessing support. Attendees of the interventions reported how these experiences helped retain engagement with the intervention, and one article (Bahu, 2019) reported the potential influence on low dropout rates.

As the interventions were delivered in the local language held by participants either through translated materials or the use of language interpreters, participants reported benefits to this in helping engagement with interventions. In all three studies, group discussions encompassed spiritual and religious aspects to promote reflection among participants with references to prophetic stories or culturally understood parables. Qualitative data in one article (Bahu, 2019) showed these techniques and approaches as beneficial in establishing a sense of cultural and collective identity following on from trauma, while the incorporation of faith and spirituality helped validate participants’ experiences and manage shame effectively.

## Discussion

This study is the first systematic review focusing on outcomes and experiences of culturally adapted assessments and varied treatments for trauma experiences among racial and ethnic minority groups. Several adaptation methods were found in this review, which included the integration of sociocultural considerations, collaboration, psychoeducation, language, cultural matching, addressing stigma, training for providers, and practical considerations. Little adaptations to assessments for trauma were found in the literature. Culturally adapted interventions can reduce PTSD symptom severity immediately by post-intervention among adults from racial and ethnic minority groups. Positive feedback was found among participants engaging in adapted interventions, and noted benefits were in help-seeking behaviors for trauma and mental health. Table 3 summarizes the critical findings of this review.

**Table 3.** Critical Findings.

Summary of Critical Findings
• A total of 21 articles were included in this review that explored experiences and effectiveness of cultural adaptations to trauma assessments and interventions for racial and ethnic minority groups. • Varied adaptation methods were found in this review, which included integration of sociocultural considerations, collaboration, psychoeducation, language, cultural matching, addressing stigma, training for providers, and practical considerations. • The evidence base exploring cross-cultural adaptations to trauma assessments for racial and ethnic minority groups remains small. • Culturally adapted interventions can reduce PTSD symptom severity immediately post-intervention among adults from racial and ethnic minority groups. • Positive feedback was found among participants engaging in adapted interventions and noted benefits were in help-seeking behaviors for trauma and mental health.

*Note*. PTSD = post-traumatic stress disorder.

Results from the meta-analyses showed significant effects of a reduction of PTSD symptoms at post-intervention for adapted interventions, however a lack of follow-up data makes it difficult to know if results were sustained over longer periods of time. Meta-analysis results suggest slightly beneficial effects on reducing anxiety severity more than depression severity for adapted interventions. Non-RCT articles that were not included in the meta-analyses mostly reported statistically significant reductions in PTSD severity among participants, which looks promising. These interventions should be further tested in randomized controlled designs to offer more conclusive understanding of intervention effects for these adapted treatments. Of the small number of non-RCT articles that reported positive intervention effects on PTSD symptoms, albeit not statistically significant among small sample sizes of participants, caution is advised in interpreting these findings. Indeed, further evaluation would be beneficial to test these interventions with greater sample sizes and controlled conditions. Future research should also report a more detailed exploration of participant’s experiences of adapted interventions, given only a handful of articles exploring this in this review.

A number of articles in the review focused on targeted interventions to support refugee and asylum-seeking populations. In line with prior literature, targeted interventions that seek to address the psychosocial needs of populations impacted by war, conflict, and persecution hold many benefits (Turrini et al., 2019). While experiences of trauma among these populations are likely to vary (e.g., post-displacement trauma, fear of persecution), this review shows adapting trauma interventions to focus on somatic and sensorial elements of trauma symptoms can cater to the diverse needs of refugee populations with trauma. Additionally, Western diagnostic labels, language, ideas, and beliefs are encouraged to be avoided. Health systems of care offered to refugees may also benefit from specialized treatments available and offered within existing community networks accessed by refugees (e.g., shelter homes). Indeed, health services and humanitarian agencies provisioned to support refugee, and asylum-seeking populations would ideally involve collaborative, multi-agency input to account for the complex and varied needs of these populations as discussed (e.g., housing, health, and legal support). While psychosocial interventions aimed to address trauma among refugee and asylum-seeking populations are necessary, simultaneously challenging oppressive systems and the sociopolitical contexts that often lead to trauma (e.g., systemic violence, ethnic conflict, or war) is also as important.

Integrating sociocultural considerations into the design and facilitation of interventions and assessments were the most reported adaptations found in this review. Several studies in prior literature have argued that Western-centered beliefs can often dominate models of evidence-based psychotherapy and treatment (Koç & Kafa, 2019; Rathod & Kingdon, 2009). This argument in theory justifies how culturally adapted interventions should incorporate the local cultural and social experiences of populations into interventions in a way to understand, validate, and work alongside the cultural needs of those accessing mental health services. In line with reviews that have adapted interventions to target depressive and anxiety disorders (Chowdhary et al., 2014; Vally & Maggott, 2015; van Loon et al., 2013), this often includes reference to culturally understood metaphors, analogies, and imagery to support therapeutic intervention. In this review, parables, culturally understood stories or folklore, imagery, and metaphors all had benefits in assisting with psychoeducation of trauma symptoms, aiding in treatment activities such as breathing or relaxation work, as well as addressing mental health stigma and shame among these populations. Emotion regulation and distress tolerance skills were supported using local philosophy (e.g., drawing on Asian philosophy, Buddhism) and mindfulness practices to help ground participants with trauma. In addition, various cultures will hold specific beliefs and ideas about how and why trauma may occur in individuals which may be rooted through religious means. Framing interventions that capture the local explanatory models of illness could therefore be a way to culturally ground interventions and have practical use in facilitating interventions with religious groups. Further, different cultural groups will have varied methods of relieving distress associated with trauma. This review shows how treatment activities that include local cultural practices to promote coping strategies may be of therapeutic use here, such as addressing coping methods such as prayer, or collectivist cultures accessing supportive methods through community systems.

Several intervention articles in this review reported advantages to culturally matching participants with facilitators with regard to demographic characteristics, often ethnicity and gender. Prior research has produced mixed findings with regard to the utility of ethnic or racial matching of facilitators of interventions and service users, with two meta-analyses suggesting cultural matching does not impact treatment engagement or outcomes (Cabral & Smith, 2011; Shin et al., 2005), and some studies reporting clinical use of cultural matching (Flicker et al., 2008; Horst et al., 2012). What is clear though among prior research, is that patient preferences for cultural matching are somewhat higher for some racial groups such as African Americans (Cabral & Smith, 2011). In this review, a large proportion of articles that involved cultural matching included target populations who had experienced interpersonal acts of violence such as sexual assault and rape. Perhaps here on a contextual level, reported benefits in this review may relate to a sense of rapport through matching that captures the racialized and gendered elements of sexual trauma; whether elements of trust and safety may in fact be easier to build on through cultural matching. This becomes more apparent given the known impact experiencing interpersonal violence can have on trauma victims trusting others (Gobin & Freyd, 2014) and navigating relationships (Bell et al., 2019). This may not be as important in the prior literature which includes findings from varied service user populations who may or may not have been exposed to trauma or sexual violence.

Of note, all service providers who were culturally matched with service users in this review did report access to clinical or case management supervision for facilitators. Important factors that underpin this decision for service providers to culturally match groups therefore must include careful consideration of the nature of trauma, cultural group, patient choice, and access to effective, culturally relevant supervision for providers. On an organizational level, an argument can be made here to therefore increase racial diversity among staff offering care in trauma services to ensure cultural matching can be met or at least be considered should patients prefer and request this.

Several interventions in the review were facilitated through group work by peer lay workers. NICE guidance does reference peer support to complement treatments of PTSD (NICE, 2018). The empirical literature has cited many benefits of peer support in supporting service users with mental health needs and has become a rapidly growing area of interest for clinical services to implement peer support among services (Repper & Carter, 2011). Given that experiencing trauma can disconnect individuals from others (Dorahy et al., 2009), and additionally induce a lack of belonging felt among victims (Ellis et al., 2015), utilizing group methods facilitated through peer support may be a strong therapeutic resource. It is possible that trained peer lay workers who were included in this review with lived experience of the needs of target populations who benefited from interventions, show some support to the importance of group-based peer support in the therapeutic management of trauma.

### Implications

Future research should detail the relative experiences of adapted interventions to infer greater knowledge of which aspects of interventions improve engagement and treatment completion. While many articles included in this review reported successful findings in achieving a reduction in PTSD and trauma symptoms among participants, crucially also understanding the subjective experiences of users would only complement future research and findings.

Additionally, it is difficult to explore potential mechanisms of change with such small relevant evidence and compare wider experiences of care in this review. It is therefore recommended that future research would benefit from inferring in greater detail the participant experiences of adapted interventions through further qualitative analysis methods, complemented with robust, RCT designs over longer follow-up periods. The lack of follow-up and downstream outcome data (e.g., social functioning, relationships) across most intervention articles in this review means it is difficult to explore whether intervention effects are sustained or extended to a clinically meaningful difference (e.g., better quality of life, functioning) over time, a crucial factor in determining the effectiveness of psychosocial interventions. Only three articles included in this review focused on cultural adaptations to the assessment of trauma among racial and ethnic minority groups, and few studies explored CPTSD and complex trauma experiences among racial and ethnic minority groups.

Assessments for trauma that lack cultural sensitivity may inadvertently neglect or misinterpret the unique challenges racial groups face when addressing their trauma needs in services. As found in this review, assessment methods that capture culturally specific expressions for trauma, such as acknowledging spiritual explanations to trauma, may mitigate overlooking the needs of service users and instead contribute to feeling misunderstood by services, a common experience reported by racial groups accessing services (Taylan & Weber, 2023). This not only may strengthen mistrust toward healthcare providers but inadequate assessments may also worsen the distress individuals are already facing, risking further iatrogenic harm from services (Faulkner et al., 2021). This largely justifies the vital research need for empirical work exploring adapted trauma assessments for racial and ethnic minority groups. See Table 4 for implications for practice, policy, and research.

**Table 4.** Implications for Research, Practice, and Policy.

Research
• While culturally adapted trauma interventions appear to be effective, greater research is needed to test interventions over longer follow-up intervals. • When developing and testing adapted trauma assessments, collaborating throughout the research process with racial and ethnic minority groups is recommended to capture unique sets of preferences, experiences, and needs of cultural groups.
Practice
• Consultations and collaboration with service user groups and community stakeholders in the development of adapted interventions and assessments are recommended, which may include establishing connections with community leaders and organizations to help practitioners deliver more culturally resonant care. • Addressing sub-group differences and the specific, tailored needs of various cultural groups is an important aspect of the adaptation process and remains an important consideration for clinical practice. This may involve personalizing assessments and interventions that integrate culturally relevant languages, practices, and values into the therapeutic process.
Policy
• National and regional health agencies should revise guidelines for trauma care to review cultural adaptations for the assessment and treatment of trauma. • Expanding efforts to fund and increase racial diversity in the mental health workforce may have both organizational and clinical benefits for mental health service providers and users. • More research funding should be considered to evaluate adapted interventions and to explore the benefits of adapted interventions. • Policies should support collaboration between mental health providers and community stakeholders to ensure that trauma care is accessible to diverse groups.

### Limitations

While there are many strengths to conducting this mixed methods review, limitations of this review exist. Interventions and assessments included in the review were developed to address different target population groups. The depth and content of the cultural adaptation processes therefore may differ between groups, due to the commonalities and differences in cultural needs across varied population groups. This additionally exposes potential challenges when comparing diverse cross-cultural studies in a meta-analysis. We also were only able to review articles published in peer-reviewed journals, reducing the scope for finding suitable material in grey literature. It is possible that information existing within the grey literature may have included applications of adapted interventions which may have been useful for the review.

## Conclusion

The evidence base showcasing the effectiveness and experiences of culturally-adapted trauma assessments and interventions remains small. This systematic review aims to add evidence to the empirical literature addressing prior gaps and methodological limitations. Results from this review highlight the importance of tailoring interventions and assessments of trauma for specific racial and ethnic minority groups. Future research should test intervention effects over longer follow-up periods for further conclusive findings around the sustainability of interventions to address trauma outcomes. Research should ensure guidance on adaptation processes is detailed for greater reciprocity of findings, and further studies exploring experiences of interventions or assessments are recommended.

## Supplemental Material

sj-docx-1-tva-10.1177_15248380251320982 – Supplemental material for Cultural Adaptations to the Assessment and Treatment of Trauma Experiences Among Racial and Ethnic Minority Groups: A Mixed-Methods Systematic Review and Meta-Analysis Supplemental material, sj-docx-1-tva-10.1177_15248380251320982 for Cultural Adaptations to the Assessment and Treatment of Trauma Experiences Among Racial and Ethnic Minority Groups: A Mixed-Methods Systematic Review and Meta-Analysis by Lewis Benjamin, Steve Gillard, Jessica Jones Nielsen, Mariana Costa E. Silva and Jacqueline Sin in Trauma, Violence, & Abuse
