# Supplementary material for: Cultural Adaptations to the Assessment and Treatment of Trauma Experiences Among Racial and Ethnic Minority Groups: A Mixed-Methods Systematic Review and Meta-Analysis
Source: Trauma Violence Abuse. 2025 Feb 27;27(3):594–610. doi: 10.1177/15248380251320982 (PMC13287536; doi:10.1177/15248380251320982)
Supplement: sj-docx-1-tva-10.1177_15248380251320982 – Supplemental material for Cultural Adaptations to the Assessment and Treatment of Trauma Experiences Among Racial and Ethnic Minority Groups: A Mixed-Methods Systematic Review and Meta-Analysis [file sj-docx-1-tva-10.1177_15248380251320982.docx]

*
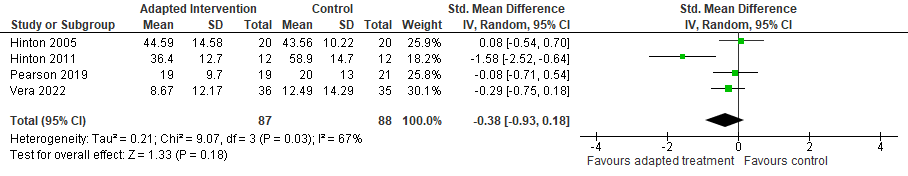
*

*Figure: Meta-analysis on participants' PTSD severity at 3-month follow up*

*
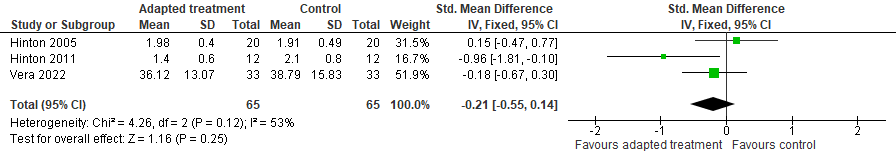
*

*Figure: Meta-analysis on anxiety severity at 3-month follow up*
